# A Novel Objective Method of Estimating the Age of Mandibles from African Elephants (*Loxodonta africana Africana*)

**DOI:** 10.1371/journal.pone.0124980

**Published:** 2015-05-13

**Authors:** Fiona J. Stansfield

**Affiliations:** The Elephant Research and Conservation Unit, Savé Valley Conservancy, Chiredzi, Zimbabwe; INRA, FRANCE

## Abstract

The importance of assigning an accurate estimate of age and sex to elephant carcasses found in the wild has increased in recent years with the escalation in levels of poaching throughout Africa. Irregularities identified in current ageing techniques prompted the development of a new method to describe molar progression throughout life. Elephant mandibles (n = 323) were studied and a point near the distal dental alveolus was identified as being most useful in ranking each jaw according to molar progression. These ‘Age Reference Lines’ were then associated with an age scale based on previous studies and Zimbabwean mandibles of known age. The new ranking produced a single age scale that proved useful for both male and female mandibles up to the maximum lifespan age of 70–75 years. Methods to aid in molar identification and the sexing of found jaws were also identified.

## Introduction

The importance of assigning an accurate estimate of age and sex to elephant carcasses found in the wild has increased in recent years with the escalation in levels of poaching throughout Africa. The MIKE (monitoring the illegal killing of elephants) and PIKE (proportion of illegally killed elephants) studies need reliable data on sex and age of found carcasses in order to predict the impact of this poaching on remaining elephant populations. The use of a simple objective field technique to estimate the age, and if possible sex, of found mandibles would help in determining the sector of the elephant population that has been removed and therefore allow a prediction of the remaining population and its structure.

In the elephant, the caudal part of the corpus maxilla of the skull contains the dental alveolus of the upper cheek teeth and the pars molaris of the mandible body carries the lower teeth. In addition to these teeth, African elephants usually carry two second incisors (tusks) that are embedded in the alveolar process of the incisive bone of the skull [1]. Elephants have no other incisors or canines in the maxilla or mandible. The elephant cheek teeth are named molars 1 to 6 (M1–M6 or MI–MVI). This acknowledges, but does not use, the palaeontological distinction between pre-molars and molars in fossil forms of proboscidia [2]. Each tooth is formed of perpendicular laminae, or lamellae of enamel filled with dentine that are held together with cementum to form a single elongated tooth. Each successive molar is larger than the previous one and may be made up of a similar or greater number of lamellae. As the tooth moves forward and upward and comes into wear at the occlusal surface, the lamellae are worn down and the ridges of enamel, cementum and dentine become progressively eroded at differing rates to form an effective ridged grinding surface. The maximum lifespan of an elephant has been disputed over the years but recent findings suggest this to be between 70 and 75 years of age [3].

Elephant mandibles have long been used to gauge the age of an individual, however, these methods have proved to be either unreliable or difficult to carry out in the field [4–11]. An accurate estimate of the age of dead elephants (obtained from their mandibles) placed together with their body measurements may also aid in estimating elephant age in the same live population based on standing shoulder heights. In addition, biological studies on elephants using post-mortem samples, or studies addressing management issues such as contraception, should be enhanced by knowing the age of the individuals. It would be useful to have a practical system of ranking elephant mandibles that could be easily used by field workers across the continent.

In the 1960s, Laws [12] and Sikes [13] independently published the results of their respective ageing studies and these have formed the basis of all ageing methods used today. The two techniques agreed that the criteria for molar identification should be based on the width and length of the full molar and the number of lamellae present, although this varied between the authors’ results. Laws [12] also stated the existence of supernumerary teeth or a 7^th^ molar. Both techniques allocated a ranked scale based on their definition of molar progression and, whereas Sikes [13] made comparisons between her age scale and zoo elephants, Laws [12] did not.

Sikes’s [13] foramen mentale (FM) technique has often been ignored subsequently owing to its lack of allocation into age groups over 30 years (the oldest zoo elephant she made comparison with was 27 years) and its dependence on a given number of lamina per molar. But despite these shortcomings, the Sikes [13] method has been expanded upon by some workers [5,6,14,15] who regarded it as being less subjective than the diagrams constructed by Laws [12].

Laws [12] divided his ranked scale of jaws into 30 groups and then applied these to an arbitrary time scale that assumed a longevity of 60–65 years. The scale was then checked and validated by comparison with other biological criteria such as shoulder height, body weight, tusk growth and the numbers of incremental layers of growth in the molar roots. The accuracy of the scale was confirmed to some extent by finding that the growth layers resulted in an overall longevity of approximately 65 years. Despite some irregularities in the Laws scale [12,16] it has nonetheless been generally adopted [4,5,7,10,17,18]. Jachmann [17] made a further comparison between the Sikes [13] and Laws [12] methods and he compared them with the findings in captive elephants [19]. From his deductions, he made various adjustments to Laws [12] ageing technique between the ages of 10 and 30 years and now research projects commonly quote elephant ages as defined by Laws [12] with Jachmann [17] adjustments.

A further detailed study [20] created a complicated ageing technique specifically designed to achieve a comparison in molar progression between the lower and upper sets of molars in extant elephants as an aid for paleontologists studying fossil proboscidean records where usually only one occlusal surface, or even a single molar is all that is available for examination.

All of the above methods for ageing African elephants are in use today. However, they all suffer from either inaccuracy or difficulty of application in the field [4–11], so that no one technique has been adopted universally. Historically, in the absence of jaws from known age wild elephants, ageing techniques have been supported by studies of other biological criteria such as eye lens weight [13,21], weight of a hind leg [22], height at the shoulder [9,13,23], back length and/or body weight [13] and foot dimensions [24,25]. The length and circumference of the tusks and epiphyseal fusion of the long bones have also been addressed [6]. Pilgrim and Western [26] inferred the sex and age of African elephants from tusk measurements made on 3000 elephants collected during the R M Laws directed culling operations in Uganda, Kenya and Tanzania between 1965 and 1969. Although they noted some anomalies in the Laws [12] ageing technique, they nevertheless assessed it to be robust in validating a process for inference of age and sex by tusk size.

A recent study of jaws collected from the wild population of elephant in Amboseli National Park in Kenya [3], has finally allowed a valid comparison of all the above methods using jaws of known age. This long-term demographic study in Amboseli NP, which began in 1972, has assigned all elephants in the population (n = 2490) a birth year. It concluded that, although the differences between tooth age as determined by the Laws technique and the age of known individuals could range from -6 to +16 years, the median difference of ± 0.5 years showed that the ageing criteria described by Laws [12] were robust in all but the 4 oldest Laws categories of the elephant in which Lee et al. [3] suggested a revision based on a maximum age of 75 years.

Now that mandibles from animals of known ages up to 40 years, and mandibles from estimated ages up to a limit of 75 years have been examined [3], it is timely to re-assess the different techniques used for ageing African elephants based on the mandible. Hence the aim of the present study was to produce a simple but robust method of objectively measuring molar progression through the elephant mandible that can be used readily by field workers. Any possible sexual dimorphism in the jaws was also examined. Following confirmation by Lee et al. [3] of the accuracy of the Laws [12] diagrams, the revised technique would be associated with their respective age data to produce a ranked scale of molar progression in the mandible which irons out the irregularities in age groups experienced when applying the Laws [12] technique alone.

## Material and Methods

Elephant mandibles (n = 323) were obtained from either professional hunting situations (in the Zambezi Valley or Hwange Safari Area), management organised culling operations (Savé Valley Conservancy) or from natural deaths on private reserves (Mwanga Lodge) between 2005–2013. Parks and Wildlife Management Authority (PWMA) gave permits to the appropriate hunters to hunt/cull the animals and the PWMA, Savé Valley Conservancy and Mwanga Lodge gave the author permission to use the mandibles. Elephants (n = 321) were shot at close range using high calibre hunting rifles and two died of natural causes. Sample collections were opportunistic, no animals were killed specifically for this study, and all permission was obtained from the relevant authorities.

**Fig 1:**
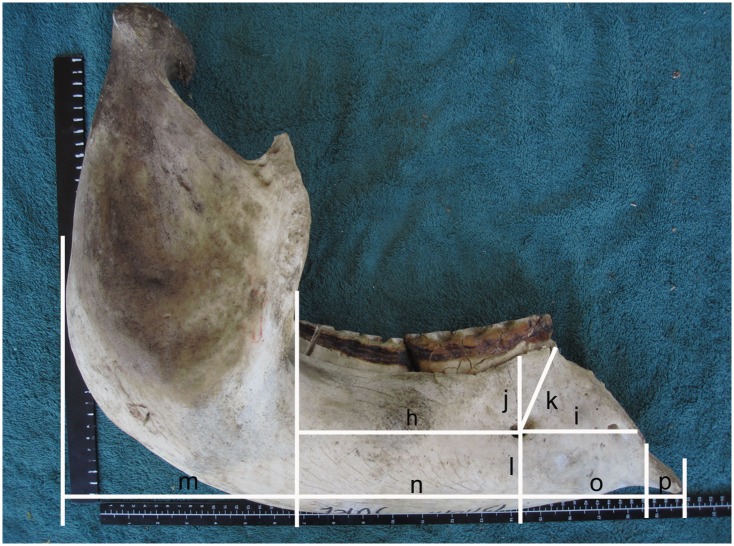
A range of measurements made on 323 intact mandibles of African elephants. The lateral surface of a bisected mandible showing attempts to describe the position of the foramen mentale (h,i,j,k,l) and measurements of the length of the jaw (m,n,o,p). Note the mandible has been rocked forward from its centre of gravity in an attempt to maintain consistency in measurement between animals. None of these measurements proved to be useful in determining a stable reference point.

Following the measurements of height, length and width (unpublished data) the mandibles were bisected at the symphysis and a further 20 records were noted on each of 272 of these mandibles (128 male and 144 female) to study the positions and number of FM (Fig 1) and to identify the location of a new reliable reference point. For these measurements, the bisected mandibular body was placed flat on a clean surface with a ruler placed below and parallel with the mandibular body and a second ruler placed at right angles to the first and touching the distal point of the mandibular ramus. These measurements included 5 on the lateral side of each mandibular body to attempt to determine the position of the FM and a further 4 measurements along the length of the mandibular body on both sides. The number of FM present on both sides of the jaw was recorded. The FM point was established as described by Sikes with adjustments by Craig and Peake [14], this being taken using a straight line from the centre of the hind-most FM to a point on the molar surface at right angles to the occlusal surface.

All mandible measurements were recorded in cm to one decimal point (using a metal tape graduated in mm) and photographed by a single recorder, as mal-alignment of the jaw during measurements could greatly influence results. Measurements on each molar and on the occlusal surface were made in cm, to one decimal place, using calipers.

**Fig 2:**
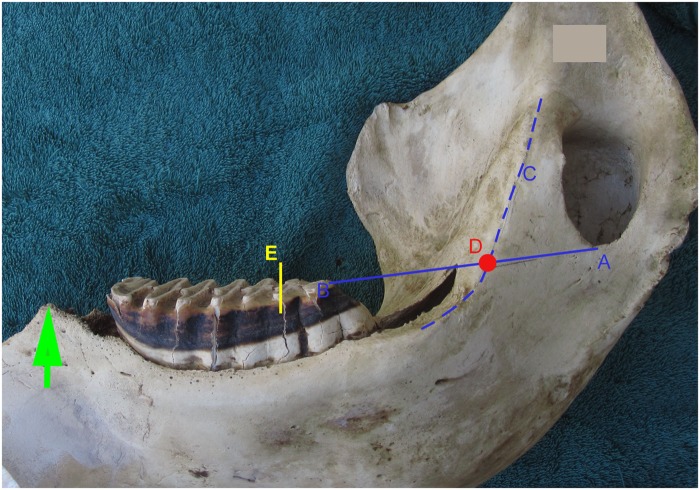
Demonstrates age estimation in the African elephant using the Age Reference Line. A straight line is drawn from Point A at the base of the mandibular foramen to Point B at the most distal point of molar occlusal wear. A line is drawn or vizualised along the ridge of the medial mandible (Line C). The two lines intersect at the red dot, Point D, which has been termed the Age Reference Point (ARP). A measurement of 10 cm is made from this point passing centrally through the distal molar. In this example 10 cm is marked as the distance from ARP (Point D) to the yellow line at E. This yellow line at E is termed the Age Reference Line (ARL). In this example the ARL falls on the 7^th^ lamella (L) of the 4^th^ molar. By reference to this ARL in Table 1 the elephant’s age can be estimated as 11.5 years. The green arrow at the mesial end of the mandible marks the mesial end of the dental alveolus, named the ‘crest’.

**Fig 3:**
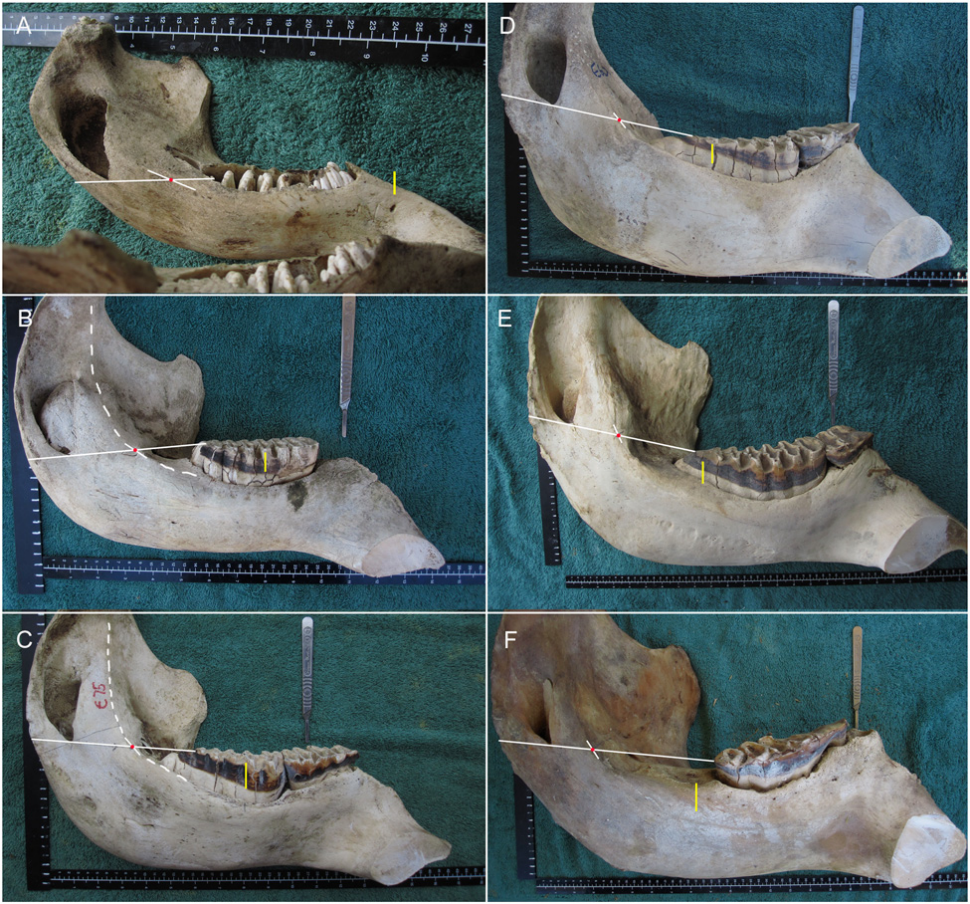
Placement of the Age Reference Point (ARP; red dot) and Age Reference Line (ARL; yellow line) on jaws of varying ages. **A)** Near-term fetus M1_–1.6 cm. **B)** A female at M3L5 (age 4 y). **C)** A female at M4L3 (age 7.5 y). **D)** A female at M5L6 (age 22 y). **E)** A male at M6L8 (age 40 y). **F)** A female at M6 + 2.3 cm (age 67 y). The dashed line (in B and C) marks the full extent of the medial ramus ridge, which is used to identify the ARP. The scalple handle indicates the placement of a foramen mentale on the lateral surface of the mandible.

**Fig 4:**
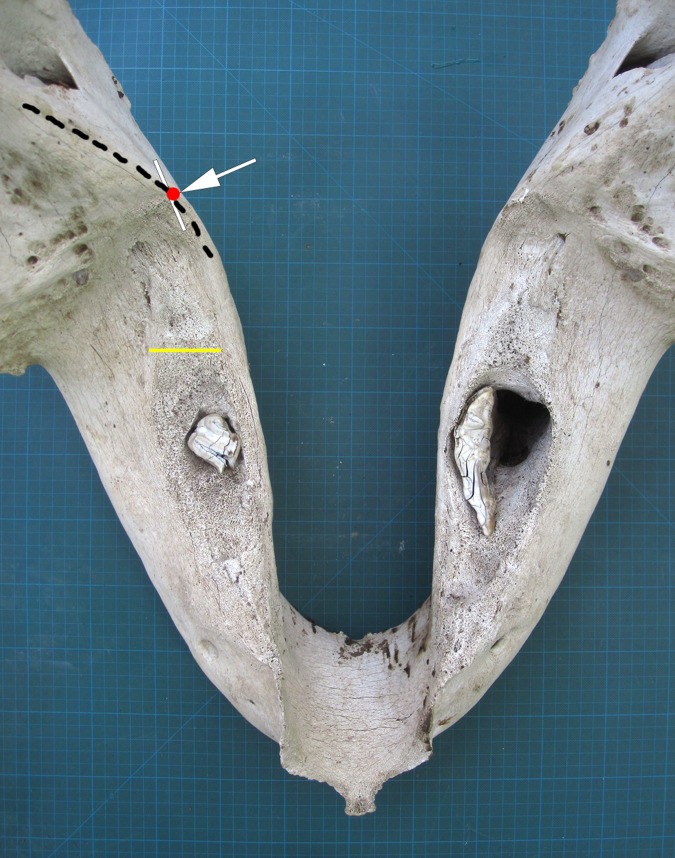
A mandible of an aged female African elephant. The white arrow/red dot marks the Age Reference Point (ARP) and the yellow line marks the Age Reference Line (ARL). The remnant of the tooth in the right mandible had rotated as shown. Grid = 1cm^2^.

A stable reference point situated close to the alveolar apex (Fig 2), proximal to the occlusal surface on either side of the jaw, was identified and termed the ‘age reference point’ (ARP; Fig 2). To determine this point, a straight line from the distal occlusal surface to the base of the mandibular foramen (MF) and continuing to the back of the ramus, was selected as a starting reference (see Fig 2; In this study, this was performed on each side of the mandible in order to gauge any possible difference between left and right). As a reference **point** was required, a line of intersection was provided using the arc formed by the ridge of the medial ramus surface (medial ramus ridge), which merges mesially into the alveolar crestal plane (see Fig 2). After many observations and measurements, it became evident that this point of intersection occurred just above the opening of the dental alveolus proximal to the occlusal surface, and it varied very little throughout life or between sexes. In order to gauge molar progression on jaws of all ages, a distance of 10 cm from the ARP was chosen to represent the Age Reference Line (ARL) beneath which the lamella of the tooth in question would be studied and described as, for example, M6L3 (third lamella of the sixth molar). An ARL at 10 cm from the ARP in a perinatal mandible fell at 1.6 cm mesial to the M1 (Fig 3A). The same mark placement fell 3.8 cm distal to the 2 cm long remnant of the M6 in a >70 year old female (Fig 4). From this rank of all the molars and their constituent lamellae Table 1 was created.

**Table 1:** Molar progression table relating age to Age Reference Line.

ARL^1^ stage	ARL age^2^ allocated (years)	Laws 1966 age class^3^	Lee et al. 2012 age class^4^	Reference to Figures and Problem areas in ageing
preM1	Fetus	I	<3m	Fig 3A
M1L1	0.12	I	<3m	
M1L2	0.24	I	<3m	
M1L3	0.36	II	0.25	
M1L4	0.5	II	0.25	
M1L5	0.8	II	0.25	
M2L1	1.0	III	1.25	
M2L2	1.1	III	1.25	
M2L3	1.2	III	1.25	
M2L4	1.3	III	1.25	
M2L5	1.4	III	1.25	
M2L6	1.6	III	1.25	
M2L7	1.8	IV	2.3	
M3L1	2	IV	2.3	
M3L2	2.5	V		
M3L3	3	V		
M3L4	3.5	V		
M3L5	4	V-VI	4	Fig 3B
M3L6	4.5	VI-VII	4–5	
M3L7	5	VII	5	Note 1
M3L8	5.5	VII	5	
M3L9	6	VII-VIII	5–7.8	
M3L10	6.5	VIII	7.8	
M4L2	7	VIII	7.8	
M4L3	7.5	VIII–IX	7.8–9.7	Figs 3C and 9B
M4L4	8	IX	9.7	
M4L5	9	IX	9.7	Fig 9D
M4L6	10	IX–X	9.7–8.5	Fig 10E
M4L7	11.5	IX–XI	9.7–14	*Note 2, Figs 2, 8A and 10F top
M4L8	13	X–XI	8.5–14	Fig 10G top
M4L9	14.5	XI–XII	14–16	Fig 10H top
M4L10	15	XI–XII	14–16	Note 3
M5L2	16	XII–XIII	16	Note 4, Fig 9A top
M5L3	17.5	XII	16	Note 4, Fig 9B mid
M5L4	19	XIII–XIV	16–20	Note 5, Fig 9C top
M5L5	20.5	XV	21.5	Fig 9D mid
M5L6	22	XIV–XVI	20–24	Note 6, Figs 3D and 10E mid
M5L7	23.5	XV–XVI	21.5–24	Figs 8B and 10F mid
M5L8	25	XV–XVIII	21.5–31.5	Figs 7A and 10G mid
M5L9	26.5	XVI–XVII	24–27	Fig 10H mid
M5L10	28	XVII–XVIII	27–31.5	Fig 11I top
M5L11	28.5	XVII–XVIII	27–31.5	Note 7
M6L2	29	XVIII–XIX	31.5–30	Fig 9A low
M6L3	30	XVII–XIX	27–30	Fig 9B low
M6L4	32	XVIII–XX	31.5–31	Fig 9C low
M6L5	34	XIX–XXI	30–35	**Note 8, Figs 7B and 9D low
M6L6	36	XX–XXI	31–35	Fig 10E low
M6L7	38	XXI	35	Fig 10F low, Fig 8C
M6L8	40	XXI–XXII	35–37	Figs 3E, 7Ci & 7Cii and 10G low
M6L9	42	XXII	37	Fig 10H low
M6L10	45	XXIII–XXV	41–48	Fig 11I low
M6L11	48	XXIV–XXVI	46.5–47	Fig 11J
M6L12	52	XXIV–XXVII	46.5–58.5	Fig 11K
M6L13/0.5cm	56	XXV–XXVII	48–58.5	
+1cm	60	XXVI–XXVIII	47–67.5	Fig 7Ciii & 7Civ
+1.5cm	63	XXVII	58.5	
+2cm	66	XXVII	58.5	
+2.5cm	68	XXVIII–XXX	67.5–68	Fig 3F
+3cm	70	XXIX–XXX	62–68	
+3.5cm	70+	XXX	62–73	

^1^ ARL class as allocated to all jaws in the study

^2^ ARL age allocated based on a best fit with the Laws [12] and Lee et al. [3] data

^3^ The Laws [12] age class was allocated to photographs of the occlusal surface of all jaws in the study

^4^ The Lee et al. [3] median age as allocated to Laws [12] criteria. Some of the classes are inverted

* No difficulty in molar identification from birth to this age. There is little difficulty in identifying molar types 1, 2, and 3 which makes ageing relatively easy up to when M4 occupies the mesial position at 10 years of age and the ARL = M4L6.

** No difficulty in molar identification from here onward. To obtain further information with regard to Table 1, refer to the Notes below and Figure references in Column 5. **Note 1**. It is usually not difficult to differentiate between M3 and M4. If there is any doubt, this can be substantiated by measuring the distance from the ARP to the crest, and the inter ramus width (see Fig 2, Tables 2 and 3). In addition, it is useful to look at the first lamella of the molar in question, the first lamellae on M3 tends to fill the full width of the tooth whereas in M4 it tends to form a pillar on the lateral side. **Note 2**. Note how much smaller M4 is compared to M5 despite there being a difference of only 1 lamellae (Fig 10F). M4s are narrower and shallower and tend to have thinner enamel ridges. The base of the root in M5 is also much longer and the gap between the roots of L3 and L4 much wider. See Fig 10G and 10H for further development of M4. **Note 3**. The Rank M4L10 rarely occurs as this lamellae is usually very small and if present becomes eroded by the mesial end of M5. None were found in this study and therefore there is no photograph. If the ARP to crest is <25 cm then it is suggestive of this being an M4. If ARP to crest >25 cm this suggests it is an M5. **Note 4**. By this stage the M5 molar is unlikely to be confused with M4 and the alveolus of M6 is usually visible, indicating that this is M5, not an M6 (this cannot be completely relied upon as 7^th^ molars do occur in a small percentage of mandibles). See Fig 9 of the development of M5 and M6 at this ARL (M5 and M6 at L stage 2 [Fig 9A] and L stage 3 [Fig 9B]). **Note 5**. The distal end of M5 is much rounder than the distal end of M6, which is more tapered throughout development. **Note 6.** From here on, male mandibles and molars are noticeably larger than females. **Note 7.** M5L11 may rarely occur, none were recorded in this study. **Note 8.** From the age of 35 (M6L6), packing bone can be seen developing behind the M6 alveolus, indicating that this is M6. If an M7 is developing they can usually be seen to be truncated and irregular in shape.

Three further measurements were then made on each mandible body; i) from the ARP to the mesial edge of the dental alveolus (termed the crest); ii) ARP to the FM line; and iii) ARP to the point of the mandibular symphysis. Bilateral ARP measurements must be taken before the lateral surface of the dental alveolus is opened, otherwise unilateral measurements can be taken when a single alveolus has been opened.

**Fig 5:**
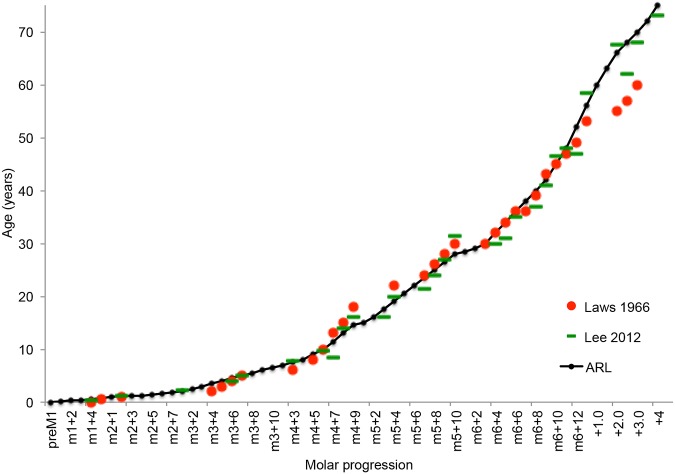
The Age Reference Line (ARL) ranking, showing the molar progression stage associated with data from previous ageing studies [3, 10]. Plateaus can be seen in the graph because some lamellae move through more quickly than others, particularly the smaller ones at each end of molars, and some lamellae may not pass the ARL in occlusal wear (eg M6L1).

For age association, all the jaws examined were initially allocated to a Laws [12] age group from photographs of the occlusal surface of the mandibles. The jaws were then re-ranked according to the newly established ARL data. Group mean data on known age jaws from Amboseli Park [3] and mean ages allocated by the Laws [12] parameters were plotted on a graph (Fig 5). A new scale of age based on the Laws [12] and Lee et al. [3] data was then plotted to match the progression of the lamina through the jaw as indicated by the ARL. When relating to elephant age, the term ‘>70’ refers to animals over the age of 70 but with a maximum age of 75 years; ageing during this period may be greatly influenced by natural variation and perhaps diet. The findings in this study were also supported by photographs of molars of live, semi-domesticated elephants of known age (± 1 year) living in Zimbabwe, South Africa and Zambia, the oldest animal being a female of 47 years of age.

For statistical analyses (Tables 2 and 3), data was compared between age groups according to molar L number, and between sexes. Data was entered in an Excel spreadsheet and analysed using Stat Plus 2009. All data met the requirements for parametric tests, therefore a t-test was used to compare two groups and one-way ANOVA was used for 3 or more groups. Results are quoted as mean ± standard deviation and n = the number of observations. The null-hypothesis between classes was rejected at p <0.05. Results where p <0.01 were considered highly significant.

**Table 2:** Measurements from the Age Reference Point to the mesial end of the dental alveolus made on African elephants in Zimbabwean populations.

Molars*	Age (y)	Male Mean ± SD	n	Female Mean ± SD	n	Between sex p-value
M1	0–0.9	13.3 ± 0.25	2	12.9 ± 0.15	3	0.130
M2	1–1.8	14.3 ± 1.00	7	13.3 ± 0.58	6	0.053
M3	2–6.5	19.8 ± 1.80	18	18.5 ± 2.39	11	0.118
M4#	7–15	22.2 ± 0.25	4	22.3 ± 3.08	30	0.920
M5	16–28.5	28.2 ± 1.74	17	26.5 ± 1.24	40	<0.001
M6	29–70+	31.5 ± 1.26	70	28.1 ± 1.23	47	<0.001

With the exception of between M1 and M2 group, significant differences (<0.001) were found between all molar male groups, all molar female groups and molar groups including totals of both male and female.

*For example, from M1L1 to M1L5 for Molar 1. For molar 2, M2L1 to M2L7. And so on for the other molars.

^#^ All 4 males were 7–8 years of age while the females range from 7–15 years.

**Table 3:** Mandible width of African elephants within Zimbabwe.

Molars*	Age (y)	Male Mean ± SD	n	Female Mean ± SD	n	Between sex p-value
M1	0–0.9	19.3 ± 1.06	2	18.9 ± 0.85	4	0.659
M2	1–1.8	24.1 ± 1.20	9	23.1 ± 1.84	7	0.226
M3	2–6.5	29.1 ± 2.95	25	28.1 ± 2.44	19	0.210
M4#	7–15	34.0 ± 3.33	6	33.7 ± 2.04	34	0.738
M5	16–28.5	43.0 ± 2.78	19	37.1 ± 2.78	55	<0.001
M6	29–70+	46.6 ± 2.91	75	39.0 ± 1.90	62	<0.001

Significant differences (<0.001) were found between all molar male groups, all molar female groups and molar groups including totals of both male and female.

*Molars relates to the ARLs for each molar, so for example, from M1L1 to M1L5 for Molar 1. For molar 2, M2L1 to M2L7, and so on for the other molars.

^#^ Data in this group is biased due to the low number of older male samples.

## Results

### Age Groups

The mandibles were divided into age groups based on the ARLs for each molar. Except for M4, there was no significant difference between the ages of males and females within a group. The difference in the M4 group was occasioned by the smaller number of older males; many males of this age having left the family groups but also being too young to be hunted as trophy animals.

### Foramen mentale (FM)

**Fig 6:**
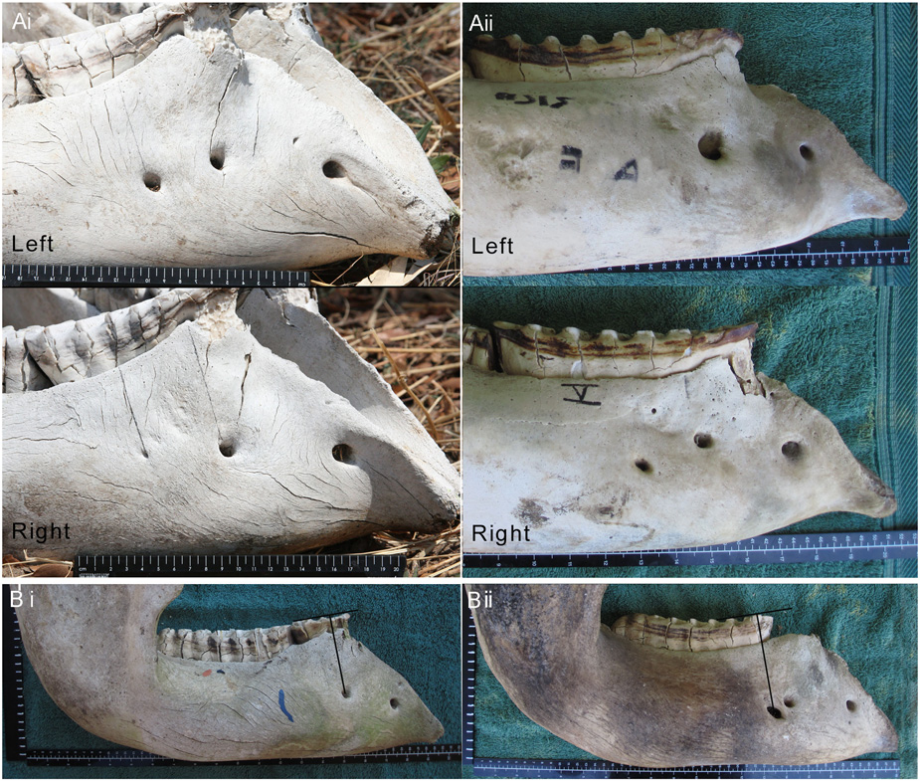
Placement of foramena mentale. (A) Placement of foramena mentale (FM) in the mandibles of two African elephants. Pictures of the left mandible are flipped horizontally to enable a comparative view of both sides of the mandible. i) Note the very small posterior FM on the right mandible compared to the left. ii) Note the variation in placement of the FM. (B) Variation in foramen mentale placement and ageing in African elephants using the FM technique. Both right mandibles of the two elephants (i and ii) have a similar Laws age but they show an age difference of 5 years when using the FM technique.

The position and number of the FM were examined to determine the reliability of the Sikes technique, which is based on the position of this nerve channel. The FM were found to vary considerably, both between animals, and between the right and left sides of the jaw of an individual and >84% of mandible bodies had either 2 or 3 FM (Fig 6A and 6B). The number of FM per mandible body ranged from 1 (2% of females and 0% males) to 5 (1.5% of females and 2% of males), and the greatest diameter from 2 mm to 45 mm, the smallest were probed using nylon line to confirm their patency. In order to maintain consistency and objectivity in the study, the rear-most FM was used as a reference point even when it was small. Furthermore, some 54% of 256 jaws showed variations in the position of the FM ageing line between the right and left sides of the mandible; 33% varied by 1 lamella, 12% by 2 lamellae, 7% by 3 lamellae, 1% by 4, and 1% by 5 lamellae. These variations were similar in male and females. Such variations, even within an animal, demonstrate that the position of the FM relative to other landmarks is not a reliable parameter for gauging molar progression between animals. A reason quoted by Sikes [13] for using the FM as a point of reference was that the roots of the molar are absorbed in the region of the FM so that the surface of the tooth anterior to the FM becomes a rootless “overhang”. In the present study however the anterior roots of the foremost molar in wear were observed to be mesial to the posterior FM in many cases (see Figs 3, 6A and 4B).

### Age reference point (ARP) and age reference line (ARL)

The ARL works on a similar principle to the FM technique [27] in identifying the lamella directly below it although the ARL has the advantage of being based on a non-subjective point at the distal end of the dental alveolus and is thereby less influenced by occlusal wear. To determine the reliability of the ARL, it was assessed on either side of 270 jaws. This revealed that 93% of these jaws produced an ARL that occurred on the same lamella of the same molar on both sides of the mandible.

The use of the ARL gives an objective indication of the progression of each molar type. Photographs of the occlusal surfaces of all 323 jaws were subsequently arranged in order of ARL, starting at Pre M1L1 and continuing through to M6L14 (see Table 1 for sequence). For older animals (aged >56) a measurement from the ARL to the distal end of the M6 was made. These images produced a single pictorial sequence or rank of molar development in both males and females from birth to old age. The ARL data was then related to Laws [12] and Lee et al. [3] age as shown in Fig 5 and Table 1 using a line of best fit.

Once the ARP had been identified, measurements from the ARP to the mesial end of the dental alveolus (termed the crest) were made on all jaws (Table 2) and this was compared with age based on all the L groups for each molar (eg. for molar 5, animals with ages marked by M5L2 to M5L11 were placed in the M5 group, etc.). With males, females and total animals, there were significant inter-group differences of the measurement from ARP to crest (Table 2) and also in the measurement of the inter-ramus width (p <0.001; Table 3). This data may also be indicative of sex, particularly from 16 years onward. Note that the findings in the M4 group have an age bias as none of the males were over 8 years of age whereas the females ranged from 7 to 15 years.

### The effect of age on the ARP

In the cohorts studied, the placement of the base of the MF from the back of the ramus, following the straight line from the occlusal surface, ranged between 2.2 cm in a late term fetus to 7.5 cm in a 58-year old bull and 7.0 cm in a >70 year old female. The distance between the base of the MF and the ARP ranged from 3 cm in a near-term fetus to 9.9 cm in cows and 10.1 cm in bulls. This indicates that, given the increase in size of the elephant mandible throughout life, the ARP remains in a relatively stable position in relation to other structures.

### The effect of sexual dimorphism on the ARL

**Fig 7:**
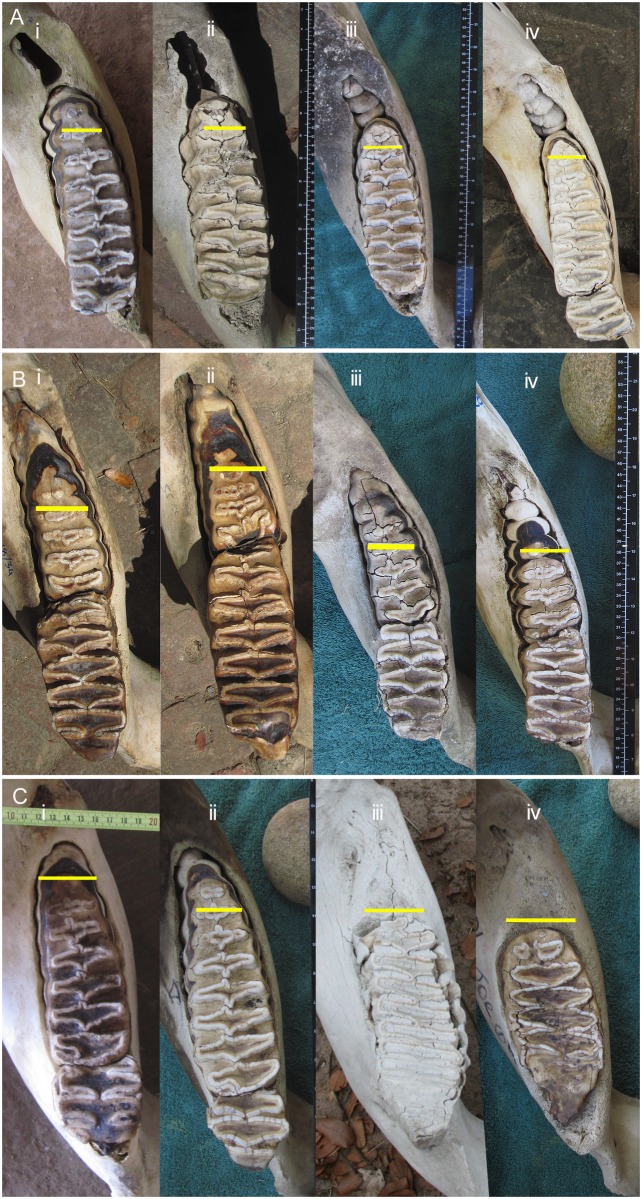
The ranking of African elephant mandibles using the Age Reference Point (ARP) and Age Reference Line (ARL) to denote molar progression, occlusal view. **A)** Four jaws at rank M5L8, i and iv are females and ii and iii males. **B)** Four jaws at rank M6L5, i and ii are male mandibles, ii and iv are female mandibles. NB The L1 of M6 is often not visible in early occlusal wear (just visible in ‘iv’) but can be identified on tooth extraction. **C)** Two jaws (i male and ii female) at M6L8, and two jaws (iii male and iv female) at M6 + 1cm. Yellow line = ARL.

Allocation of the ARL on both male and female jaws resulted in similar allocation of Laws [12] classes (Fig 7). This lack of sexual dimorphism is related to the length of the dental alveolus, the size of the molars, the distance from MF to ARP, the number of lamellae per molar class and the standard use of a 10 cm measurement to give the ARL.

### Use of photographs to aid in molar identification

**Fig 8:**
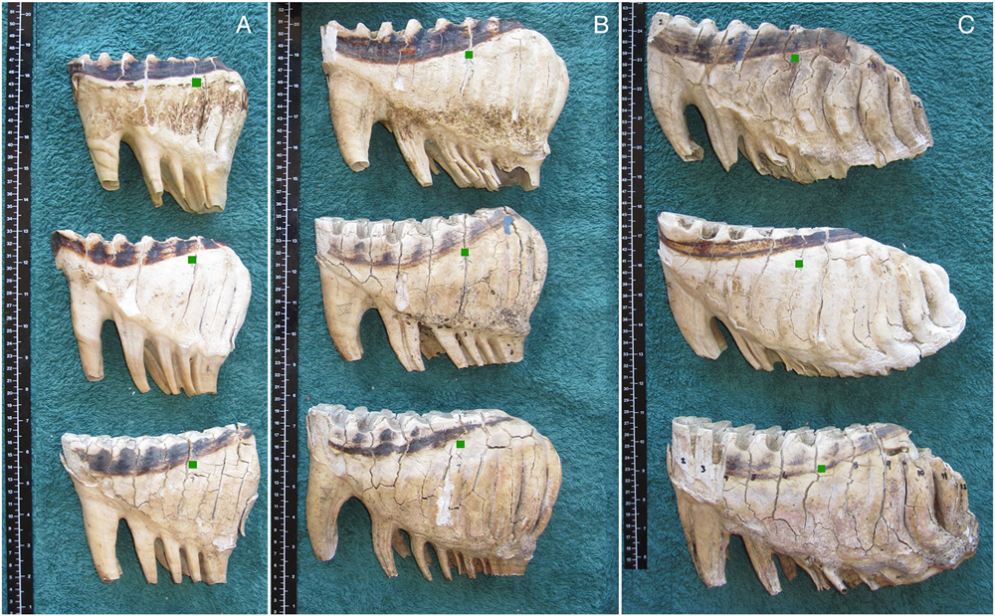
Examples of molars of African elephants at different Age Reference Line stages. Green squares indicate the seventh lamellae (L7). **A**) three molars at stage M4L7 (molar 4 lamella 7), **B**) three molars at M5L7, and **C**) three molars at M6L7. All show similar development, but not necessarily wear, in each tooth at each stage.

**Fig 9:**
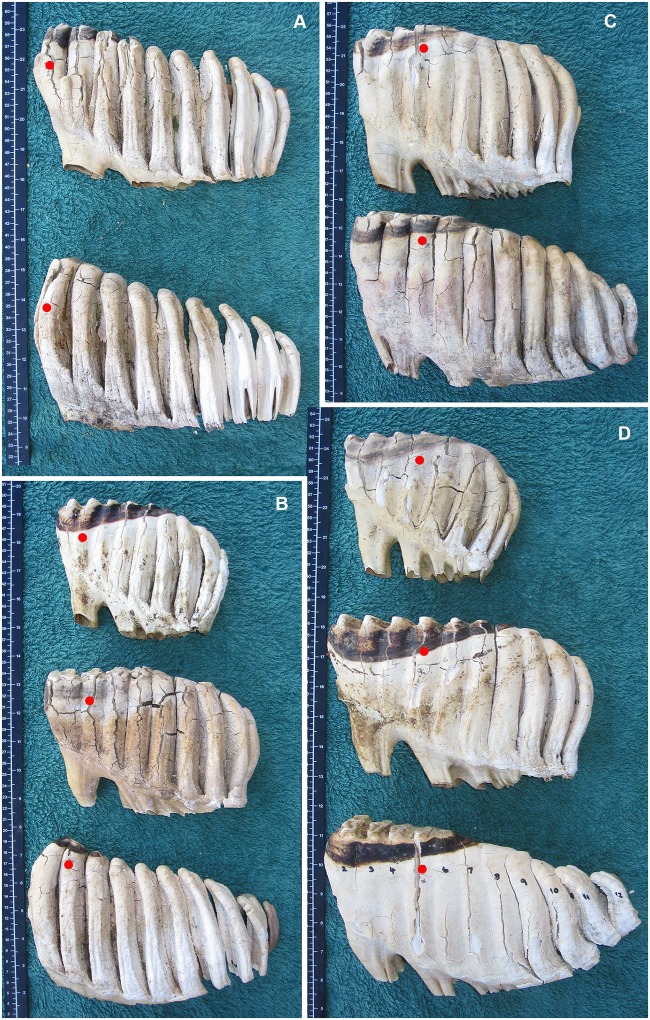
A comparison of the typical development (not necessarily occlusal wear) of molar teeth of African elephants at different lamellae numbers according to the position of the Age Reference Line. Molars are aligned with mesial end to the left with the smaller molar at the top, progressively getting larger downward. Red dots indicate the lamellae number (L), which is 10 cm mesial from the Age Reference Point (See Fig 2). A) M5 (top) and M6 (bottom) at L2. M5 tends to come into wear first on L1. Due to space restrictions, M6 tends to emerge into wear from underneath the final lamellae of M5, it therefore most commonly wears first on either L2 or L3. B) M4, M5 and M6 at L3. The root base is much longer in M5 than M4, M5 is also longer and deeper. When comparing M5 and M6, M6 is typically showing first wear on L2 or L3. In M6 the lamellae also tend to taper to a smaller size whereas M5 tends to have a convex distal end. C) M5 and M6 at L4 (no M4 available). Note the convex distal end of M5 compared with the tapered end of M6. D) M4, M5 and M6 at L5.

**Fig 10:**
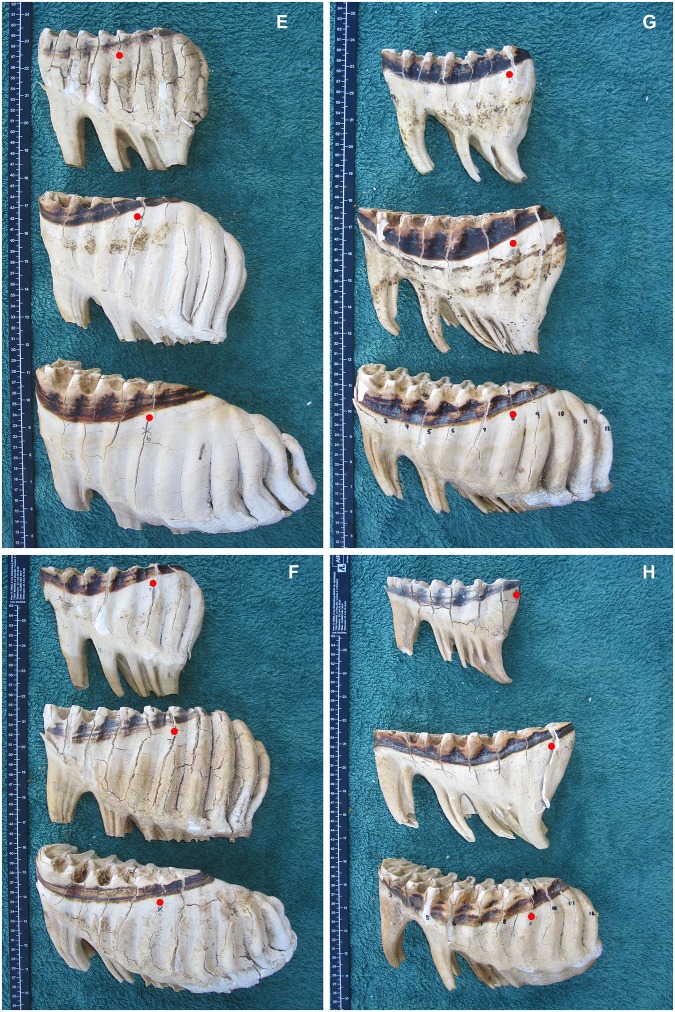
A comparison of the typical development (not necessarily occlusal wear) of molar teeth of African elephants at different lamellae numbers according to the position of the Age Reference Line. Molars are aligned with mesial end to the left with the smaller molar at the top, progressively getting larger downward. Red dots indicate the lamellae number (L), which is 10 cm mesial from the Age Reference Point (See Fig 2). E) M4, M5 and M6 at L6. F) M4, M5 and M6 at L7. G) M4, M5 and M6 at L8. H) M4, M5 and M6 at L9.

**Fig 11:**
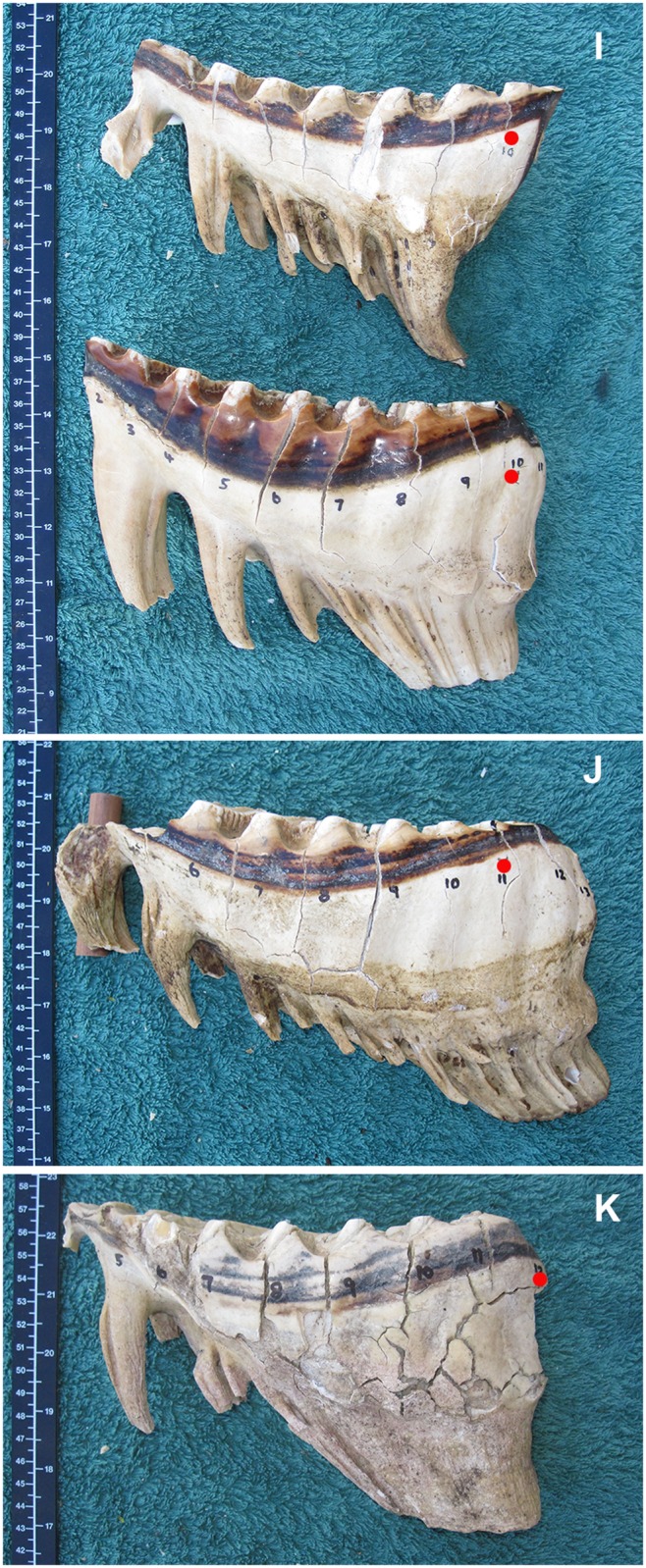
A comparison of the typical development (not necessarily occlusal wear) of molar teeth of African elephants at different lamellae numbers according to the position of the Age Reference Line. Molars are aligned with mesial end to the left with the smaller molar at the top, progressively getting larger downward. Red dots indicate the lamellae number (L), which is 10 cm mesial from the Age Reference Point (See Fig 2). I) M5 and M6 at L10. J) M6 at L11 The foremost part of this tooth is broken off and a wooden wedge is seen below the tooth. K) M6 at L12.

Groups of molars at similar ARL may be compared using photographs such as those in Fig 8 which demonstrate that there is little variation in the development of a given molar at a given age, although occlusal wear of the molar may vary between elephants. Likewise photographs of similar lamellae rank on different molars may be viewed for comparison, as in Figs 9–11, which shows typical development of molar types 4, 5 and 6 at different stages.

## Discussion

During this study the ARP was identified as an objective anatomical reference point from which molar progression in the African elephant could be measured consistently throughout life. From this reference point the ARL was developed and associated with age data from previous studies [3,12] to enable the establishment of a new ageing table for African elephants (Table 1). Although Lee et al. [3] identified natural variation in molar development between animals of the same age, the ARL does nevertheless, in the populations studied, appear to improve on previous subjective ageing estimates.

The mandibles examined in this study were placed initially in Laws [12] age categories and, in agreement with the findings of other authors [3,17], intermediate categories were found to be missing and inverted categories were observed under the age of 30 years. In this respect the Sikes [13] criteria appeared to describe a more continuous series of developmental stages.

### Irregularities with the Sikes FM technique

Throughout Sikes’s detailed studies on ageing [13,27,28] the FM was suggested to be a single nerve hole present on the buccal surface of the mesial mandibular body with a ‘divided FM’ occurring as an ‘occasional variant’ [29]. In the current study divided foramena were identified in the majority of jaws (> 98%), there being from 1 to 5 present on each mandibular body (Fig 6A and 6B). Divided FM therefore appear to be the norm in Zimbabwe and they have been noted throughout Africa in both extant and extinct proboscideans [1,30]. Hence if the FM are to be used for ageing purposes, objective decisions are required concerning the implementation of the technique. Sikes herself refers to three methods of implementation; the observation of the lamina ‘above the most posterior opening’ [29], of the lamina ‘directly above a midline drawn between the two openings’ [31] and alternatively as a vertical line through the FM [13] or a point directly above the FM [27]. Other authors have referred to the ‘main nerve channel’ [14] and to the placement of a line from the FM at right angles to the occlusal surface to denote the lamina in question. In addition, and perhaps of more concern, is the variation in the placement of the FM on either mandible body. As sources of error in measuring elephant jaws are inherent owing to its morphology [3], it was initially difficult to describe the position the FM for inter-animal comparisons. Therefore, it was decided to observe the molar progression results as determined by the FM technique between the two sides of individual jaws. This leads to different ages being allocated to the same elephant in 54% of cases based on the left and right sides of the mandible. Such variation within an animal may be predicted to also be present between different animals of the same age. Owing to the above difficulties it is suggested that the use of the Sikes [13] FM technique lacks the objectivity and accuracy attributed to it.

### Difficulties with the Laws [12] technique

Laws [12] divided his observed molar sequence into 30 arbitrary age categories that may well be distributed disproportionately relative to actual molar progression rates. When working with the Laws [12] diagrams many mandibles displayed the characteristics of more than one of the 30 proscribed classes and some clearly lay between them. In addition, cyclical peaks and troughs of birth rates have been noticed when using the Laws method and have been attributed to artefacts caused by slight inaccuracies in the age determination technique [16]. Hence attempts have been made in the current study to iron out such irregularities and to extend Laws age scale from a maximum age of 65 to >70 years (with a potential maximum age of 75) in line with the findings of Lee et al. [3].

### The age reference point (ARP)

Molar progression is suggested to be an intrinsic process, which occurs at approximately the same rate in elephants of similar age. Molar wear at the occlusal surface is related to molar progression but it is open to greater variability because it is influenced by the angle of the tooth and the balance of erosion between the upper (convex) and lower (concave) molars. For these reasons it has been proposed previously [32] that study of the eruption of the molars distally, rather than concentrating on occlusal wear, may provide a more useful analysis. Therefore a reference point in the proximal part of the dental alveolus was the target for a new objective ageing technique.

A search of human and veterinary dental literature stimulated the investigation of the mandibular foramen as a possible reference point. This anatomical feature is widely discussed in dental literature owing to its importance for inferior alveolar nerve anesthesia and dental surgery [33]. In humans, the position of the foramen seems to show symmetry between the right and left rami. Position also appears to be determined by the size, width and height of the mandible, being more posterior in a wider ramus [34]. However, there are few age-related changes in the relative position of the MF, particularly anterio-posteriorly from the alveolar crestal plane [35]. Size also exerts little influence on the position of the MF relative to the occlusal plane of the molars, where it is either level or slightly below [33], tending to be lower in children [34]. These findings are consistent with those found in the elephant mandible (see Fig 3).

A further benefit of the ARL technique is that by studying the back of the mandible, where there is limited impact of molar wear, it allows easier identification of the lamina in use as the majority of the molar in question is still present. Only on the last lamellae of M6 does the issue of number of anterior abraded and lost lamellae become a problem in lamella identification. On all other teeth, the number of a particular lamella can be gauged either by the number of lamellae present, or the root structure of the tooth (according to the placement of L3/L4 above the anterior root division).

### Why does the ARP-ARL work for all age groups and both sexes?

Logically it could be assumed that the ARL placement should diverge for males and females owing to the greater size of the male mandible after approximately age 20. This was found not to be the case and a number of factors may contribute to this:

1) The small variation in distance from MF to ARP between males and females throughout life. 2) The standard 10 cm distance from the ARP to the ARL. This distance is not enough to reflect the significant differences in the size of male molars, the length of the lamellae (mesial to distal) that form the tooth, or the increase in size of the dental alveolus. 3) During the wear of M5 and M6 in males, at an age when sexual dimorphism is highly significant, the teeth increase in size in proportion to the increase in size of the dental alveolus, the latter being 11% longer in males than in females during the wear of both M5 and M6, compared with the M5 molars being 4% longer and M6 molars being 16% longer (unpublished data). 4) The use of the medial ramus ridge line to intersect with the molar to MF line helps to negate any difference in ARP position between male and female jaws in animals of the same age.

The ageing results were found to be consistent with the mandibles of 2 Zimbabwean elephants of known age (26 and 28 years) collected at post mortem. In addition, photographs of the molars of live domesticated elephants of known age up to 47 years (± 1 y) in Southern Africa, tend to agree with the ageing criteria (it not possible to use the ARL technique precisely on live elephants). Interestingly the photographs of mandibles aged 27.2 and 27.5 years described by Lee et al. [3] which differed by 4 years with the Laws [12] diagrams, fall within the developmental range associated with ARL M5L10 (i.e. 28 years). The age class below for M5L9 is 26.5 years.

### Ageing the mandibles of elephants >50 years

Lee et al. [3] suggested an extension of the estimated maximum age of African elephants from 65 to 75 years and proposed that this should take place by expanding Laws [12] class XXVII (age 53 ± 2 y) onwards. However three factors argue that this expansion of the Laws classes should logically begin at stage XXVI instead of stage XXVII; i) the inversion of the mean Lee et al. age in Laws class XXV and XXVI, ii) the large apparent difference in molar development and wear between the Laws classes XXVI and XXVII and, iii) if the elephant’s maximum lifespan is indeed 75 years, the remaining M6 needs to last for a further 25 years at rates of erosion which are consistent with earlier life. The Lee et al. study described known-age elephants up to 40 years old exhibiting mandibles that corresponded with the Laws age classes XXII and XXIII. Further confirmation of molar progression was provided by photographs of the molars of a 47 ± 1 year old domesticated elephant in Zimbabwe, the molars of which represented either XXIII or XXIV of Laws classes; that is, 9 lamellae of M6 in wear, the first two being confluent with erosion of the distal border, it was not practical to say how many lamellae remain to erupt.

The M6 does continue to progress mesially up to extreme old age even though there is no pressure from a developing tooth behind it. Therefore at ages >56 (when the terminal lamella of M6 has passed the ARL) the ARL age is estimated by measurement between the ARL and the distal end of M6, this being at a maximum of 3.5 cm in a very aged female (Fig 4). From the small numbers of animals studied in this age group (11 females and 5 males), and there being no mandibles of known age for comparison, the M6 forward progression and the volume of tooth remaining may continue to be problematic in ageing elephants >60 years.

### Practical field use of the ARL Table for ageing elephants

The ARL in Table 1 is simple to use (see Fig 2 for method), it requires: 1) marking the ARP on the mandible in question and then 2) measuring 10 cm from this to mark the ARL on the molar in question and then 3) the identification of the number of the lamellae (L) lying under this line (lamellae being counted in sequence from the mesial end of the molar). Once the ‘L number’ of the molar is known, this can be used to identify the molar number if there is any doubt in this area (see Figs 9–11). The size of the mandible can also be helpful in determining the molar in use. The width of the mandible at each molar stage is given in Table 3, and Table 2 shows the differences found in the distance between ARP and the crest.

Having a complete set of molars, both male and female is useful and can greatly aid in molar identification.

### Conclusion

The Age Reference Line provides a useful and objective means of placing elephant mandibles in a consecutive series that indicates molar progression throughout life. This was trialled on two Zimbabwean elephant populations and an age scale based on the data of Laws [12] and Lee et al. [3] was associated with the ranking. Comparison with a further 8 mandibles from the elephant population of north-west Zimbabwe suggested that this technique would also be useable there. This is a significant finding as it is widely thought that the elephants of the north-west are much taller than those of the north and east. Indeed they are some of the tallest elephants in Africa [6,9,25,36] and might therefore have had larger mandibles. Hence, if the ARL technique is valid within the range of elephant statures in Zimbabwe, it is likely to be useful across Africa. New methods to aid in molar identification and sexing of found mandibles have also been suggested.

Further studies are planned to investigate the use of the ARL technique on other elephant populations in order to address the potential impacts of a variable number of lamellae per molar, mandible size and diet.
